# Esophageal achalasia, diagnosed through the repeated manometry, alleviated using benzodiazepine: A case report

**DOI:** 10.1097/MD.0000000000033494

**Published:** 2022-04-07

**Authors:** Ryusei Nishi, Haruka Amitani, Kazumasa Hamada, Takamasa Fukumoto, Ryuichi Kato, Takako Yamamoto, Yuuki Fuku, Kenichiro Sagiyama, Akihiro Asakawa

**Affiliations:** a Department of Psychosomatic Internal Medicine, Kagoshima University Graduate School of Medical and Dental Sciences, Kagoshima, Japan.

**Keywords:** case report, dysphagia, esophageal achalasia, esophageal manometry

## Abstract

Idiopathic achalasia is an esophageal peristaltic dysfunction of the lower esophageal sphincter (LES). The initial symptom is progressive dysphagia. However, due to its rarity, it is often misdiagnosed as an esophageal disorder. High LES pressure on esophageal manometry is an essential finding for the diagnosis.

**Patient concerns:**

A 55-year-old man was hospitalized with saliva-like vomitus, stuck-in-throat feeling of dysphagia, and weight loss.

**Clinical findings:**

On initial admission, gastrointestinal endoscopy, esophageal manometry, laboratory tests, and physical examination results were within normal limits.

**Diagnoses, interventions, and outcomes:**

Initially, the patient was diagnosed with globus sensation and recovered with medication. However, the symptoms recurred. He requested another examination on the second admission and was diagnosed with achalasia based on repeat esophageal manometry. The patient recovered after surgical treatment.

**Lessons:**

When patients still suffer from these symptoms, there is a need to reconsider achalasia, even if it is initially excluded from the differential diagnosis. Medication is not a radical treatment; however, it sometimes ameliorates symptoms. Moreover, the psychosomatic approach can be useful in such cases.

## 1. Introduction

Idiopathic achalasia is a primary dysfunction of esophageal peristalsis and insufficiency of lower esophageal sphincter (LES) relaxation. The initial symptom is progressive dysphagia.^[1]^ The annual incidence is reported to be approximately 1 per 100,000 people.^[1,2]^ Due to its rarity, it is often misdiagnosed as an esophageal disorder. Diagnostic testing of achalasia includes endoscopy, radiography, and esophageal manometry. Definitive treatments include pneumatic dilation, laparoscopic Heller myotomy, and peroral endoscopic myotomy (POEM). Therefore, pharmacotherapy is not the first-line treatment.^[3]^ We present a case of achalasia with primary normal but secondary abnormal findings on esophageal manometry, which showed efficacy with anxiolytic agents before undergoing POEM.

## 2. Patient information and clinical findings

Informed consent from the patient was unavailable; however, the institutional review board takes responsibility for the anonymization of the patient. This study was approved by the institutional review board of Kagoshima University Hospital (number 220263).

A 55-year-old man was hospitalized for saliva-like vomitus, stuck-in-throat feeling of dysphagia, and weight loss. The symptoms continued for a year, approximately once every 5 days. On a symptomatic day, the patient ate almost nothing. This resulted in the loss of body weight from 76 to 64 kg. Before visiting our institution, he had visited 4 hospitals, consulting different doctors, including otolaryngologists and gastroenterologists. No abnormalities were observed. At our institution, gastrointestinal endoscopy revealed that the esophagogastric junction was not obstructed, and the lower third of the esophagus spasmed intensely. Esophageal manometry showed a weak but within-normal limit primary peristaltic wave in the middle thoracic esophagus. No other abnormalities were observed. The laboratory and physical examination results were normal.

## 3. Diagnostic assessment and therapeutic intervention

Esophageal motility disorders, such as achalasia and diffuse esophageal spasm, were excluded because of the normal findings on gastrointestinal endoscopy and esophageal manometry. Moreover, there were no other characteristic symptoms, such as chest pain, heartburn, or regurgitation. Although his body weight had decreased, laboratory and physical examinations did not suggest malignancy or infectious diseases. Therefore, initial diagnosis was globus sensation. The patient recovered after 5 weeks of treatment with medication (ethyl loflazepate 2 mg/d and sulpiride 2.5 mg/d). After 4 weeks, his symptoms recurred. His symptoms were relieved after 5 weeks of hospital treatment with the same medication, but he did not completely recover before discharge. The patient requested another detailed examination. After a month, all examinations were repeated. Esophageal manometry findings were consistent with achalasia.

## 4. Outcomes

He was finally diagnosed with achalasia and fully recovered after POEM.

## 5. Discussion

The present case had 2 crucial findings. Even if one-time esophageal manometry shows normal findings, achalasia should be considered if the patient still complains of symptoms. Several studies indicate that more than 10 mm Hg LES pressure on esophageal manometry can identify achalasia with approximately 100% sensitivity.^[4,5]^ However, most esophageal manometry studies suggest that achalasia accompanies aperistalsis. Eckardt et al^[6]^ concluded that diagnostic lag results from overlooking typical findings rather than atypical clinical presentation. Although endoscopy shows normal findings, such lags can decrease if multiple physicians review radiographs and esophageal manometry is performed, even if the review of radiographs is incompatible with achalasia.^[6]^

Dysphagia without abnormal findings is often treated as a globus sensation, which causes a diagnostic lag if the patient’s actual diagnosis is achalasia. In the present case, the patient underwent radiography, endoscopy was performed more than once by multiple physicians, and esophageal manometry was not compatible with achalasia. Based on our research, there are no reports of atypical clinical presentations with little possibility of a physician-made diagnostic lag. This case highlights the importance of performing additional esophageal manometry in patients incompatible with the diagnosis of achalasia.

Pharmacotherapy is not the first-line treatment to decrease LES pressure.^[3]^ However, an important finding is that medication can be a viable treatment option for some patients. LES pressure can be decreased using benzodiazepines because they cause smooth muscle relaxation.^[7]^ However, benzodiazepines were prescribed as an anxiolytic in this patient. No study has addressed the use of anxiolytic agents or antidepressant drugs as treatment for achalasia.^[8]^ Nevertheless, the present case suggests that they can be used for treating achalasia.

Psychosomatic approaches toward achalasia need to be more focused because anxiety and stress probably delay the recovery of symptoms, and stress might exacerbate or induce achalasia.^[9]^ Also, social isolation, such as the coronavirus disease-2019 lockdown, affects patients with achalasia.^[10]^ Very few reports take a psychosomatic approach to achalasia, contrary to its importance.

In conclusion, the present case highlights the importance of reconsidering esophageal manometry for nonachalasia patients if their symptoms persist, and psychosomatic approaches, including medication, can be useful in patients with achalasia.

## Author contributions

**Conceptualization:** Ryusei Nishi, Haruka Amitani, Takamasa Fukumoto, Akihiro Asakawa.

**Investigation:** Ryusei Nishi.

**Supervision:** Haruka Amitani, Akihiro Asakawa.

**Writing – original draft:** Ryusei Nishi.

**Writing – review & editing:** Ryusei Nishi, Haruka Amitani, Kazumasa Hamada, Takamasa Fukumoto, Ryuichi Kato, Takako Yamamoto, Yuuki Fuku, Kenichiro Sagiyama, Akihiro Asakawa.
